# 287. Fecal Microbiome and Microbial Metabolites Predict Postoperative *Enteroccoccus* and *Enterobacterales* Infections in Liver Transplant

**DOI:** 10.1093/ofid/ofad500.359

**Published:** 2023-11-27

**Authors:** Christopher Lehmann, Nicholas Dylla, Matthew Odenwald, Emerald Adler, Eric Pamer, Aalok Kacha

**Affiliations:** University of Chicago, Chicago, Illinois; University of Chicago, Chicago, Illinois; University of Chicago, Chicago, Illinois; University of Chicago, Chicago, Illinois; University of Chicago, Chicago, Illinois; University of Chicago, Chicago, Illinois

## Abstract

**Background:**

Liver transplantation (LT) is associated with postoperative infections caused by antibiotic-resistant bacterial pathogens that reside in the intestine. An intact intestinal microbiome suppresses expansion of enteric pathogens, however patients with severe liver disease often have reduced microbiome diversity and increased density of antibiotic-resistant *Enterococcus* and *Enterobacterales* species. Experimental models have demonstrated that metabolites produced by the intestinal microbiome, including short chain fatty acids (SCFAs), secondary bile acids and indole compounds, enhance host epithelial and immune defenses against enteric pathogens. Microbiome derived metabolites likely contribute to resistance against infectious diseases in LT patients, however, this remains uninvestigated.

Graphical Abstract

**Figure:**
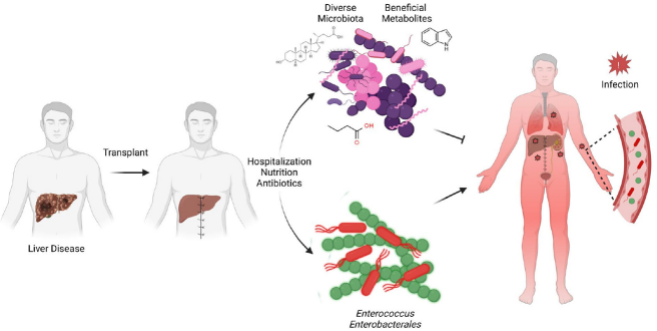

**Methods:**

We prospectively enrolled 158 liver transplant candidates and determined peri-transplant fecal microbiome compositions including relative and absolute fecal metabolite concentrations. We then used microbiome composition characteristics and metabolite profiles to predict postoperative bacterial infection.

Enrollment

**Figure:**
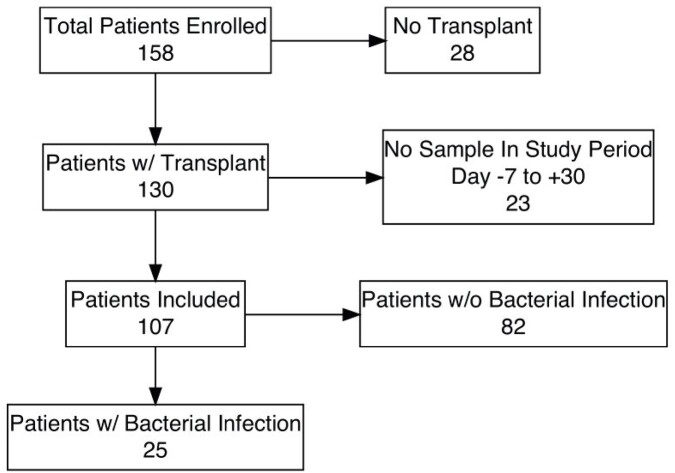

**Results:**

Fecal microbiomes in LT recipients ranged from highly diverse to complete loss of diversity resulting in expansion of *Enterococcus* and/or *Enterobacterales* species that were associated with postoperative infection. Gas chromatographic (GC-) and liquid chromatographic (LC-) Mass spectrometric analyses revealed decreased concentrations of SCFAs, secondary bile acids, and indole compounds in fecal samples with low microbiome diversity and associated expansion of *Enterococcus* and *Enterobacterales* populations. *Enterococcus* and *Enterobacterales* expansion at 20% and 2.5% respectively reliably predicted postoperative infection. Reduced short chain fatty acids, secondary bile acids, and indole metabolites predicted postoperative bacterial infection as well as *Enterococcus* and *Enterobacterales* expansion.

Microbiome Compositions of LT recipients vary widely

**Figure:**
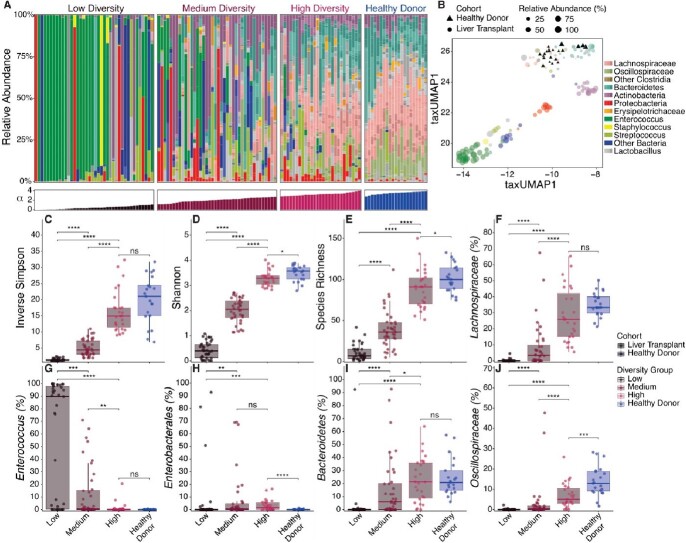

(A) Fecal microbiome composition plots of liver transplant (LT) patients and healthy donors (HD) vertically organized by relative abundance and color coded by taxa. Individual samples were ordered horizontally by Shannon diversity. (B) taxUMAP1 plot of taxonomic composition, HD are denoted as triangles. Samples are color coded by the most abundant taxon and size determined by the relative abundance of that taxon. (C-E) Comparison of Alpha diversity between LT diversity groups and between high diversity and HD using (C) Inverse Simpson, (D) Shannon, and (E) Richness. (F-J) Comparison of relative abundance of select taxa between LT diversity groups and HD. (F) Lachnospiraceae; (G) Enterococcus; (H) Enterobacterales; (I) Bacteroidetes; (J) Oscilospiraceae. Significance tested by Kruskal-Wallis test *p≤ 0.05 **p≤0.01 ***p≤0.001 ****p≤0.0001

Qualitatively measured microbiome derived fecal metabolites vare widely among LT recipients

**Figure:**
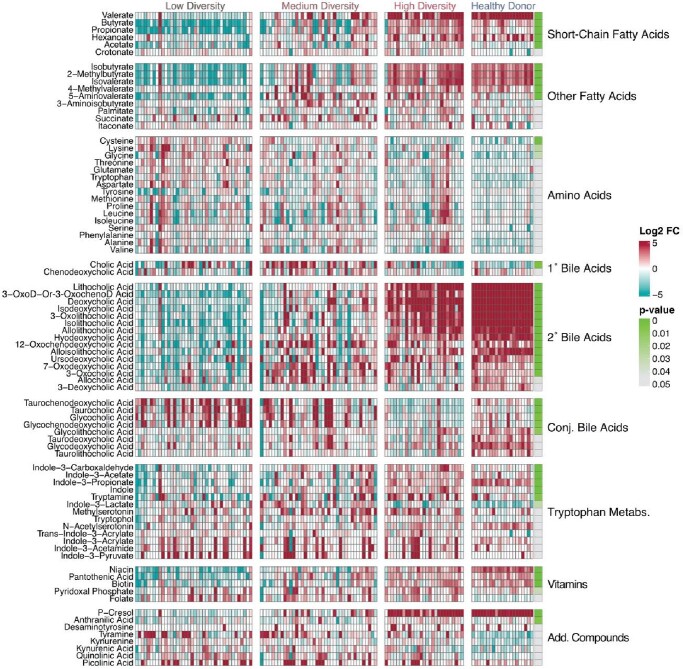

Individual metabolite abundances represented on a colorimetric heat map by log2 fold change from the mean between samples. Red indicating increased abundance; blue indicating reduced abundance. Significance was measured between LT groups using the Kurskal-Wallis test and denoted on a colorimetric scale where green represents lower p-values, adjusted for multiple comparisons. HD were included for visual comparison. Add. Compounds include kynurenine pathway and phenolic aromatics. Abbreviations: 1°-Primary; 2°-Secondary; Add.-Additional; 3-Oxo-D-Or-3-Oxocheno.-1-Oxo-Deoxycholic or 3-Oxochenodeoxycholic acid, which could not be completely discriminated chromatographically and are included together.

Enterococcus and Enterobacterales expansion microbiome predicts postoperative infection

**Figure:**
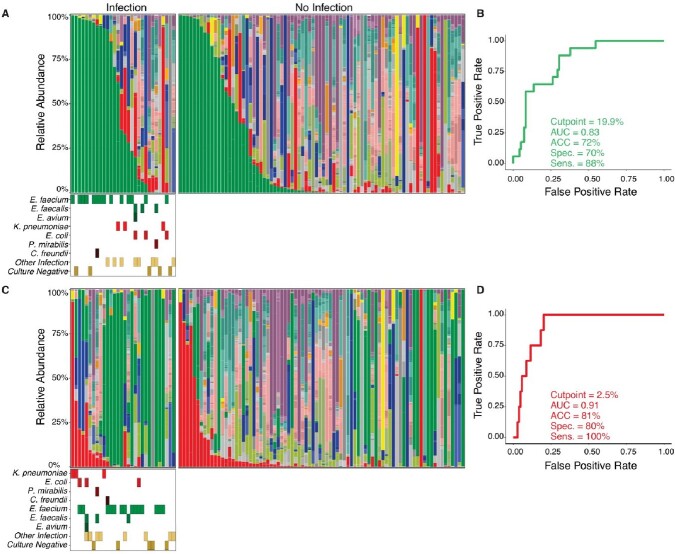

(A) Fecal microbiome composition plots color coded by taxon. Plots are categorized by presence of bacterial infection and ordered by descending relative abundance of Enterococcus. Colored tiles indicate an infection caused by the denoted organism associated with that stool sample. For taxonomic color palate refer to Figure 1. (B) Receiver operator curve using Enterococcus abundance to predict Enterococcus infection. Cut point determined by Youden Index to optimize both sensitivity and specificity. 95% Confidence intervals for Accuracy (0.74-0.91), Specificity (59-94%), and Sensitivity (65-100%). (C) Fecal microbiome composition plots organized by relative abundance of Enterobacterales. (D) Receiver operator curve using Enterobacterales abundance to predict Enterobacterales infection. 95% Confidence intervals Accuracy (0.82-0.96), Specificity (72-94%), and Sensitivity (100-100%). Abbreviations: AUC-Area under the curve; ACC-Accuracy; Spec.-Specificity; Sens.-Sensitivity.

**Conclusion:**

*Enteroccoccus* expansion, *Enterobacterales* expansion, and microbial metabolite abundances accurately predicted LT patients who developed postoperative infection.

Microbiome derived fecal metabolites identify alpha diversity and post operative infection

**Figure:**
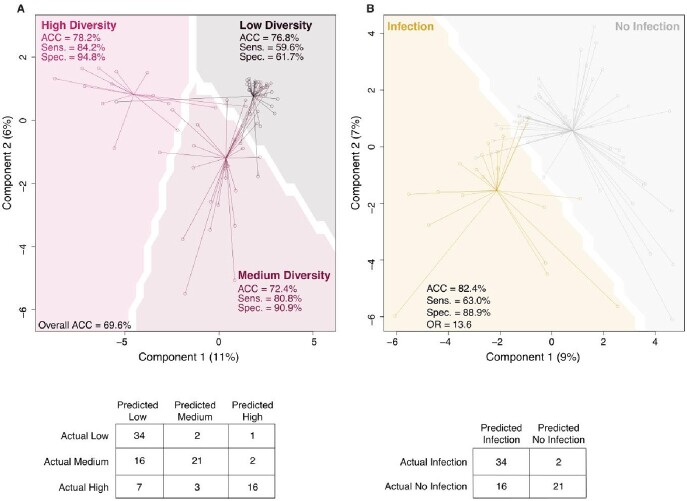

(A) sPLS-DA using input matrix of sample metabolites and predicted microbial diversity group. Comparison between predicted groups was visualized on a grid with dividing lines and optimized by maximum distance between groups. Accuracy was: Low Diversity 77%, Medium Diversity 72%, and High Diversity 78%. Sensitivity ranged from 60-84%. Specificity ranged from 62-95%. (B) sPLS-DA using input matrix of sample metabolites and predicted postoperative infection. Comparison between outcomes was visualized on a grid with a dividing line and optimized by maximum distance between groups. Accuracy was 82.2% [73.9-89.1%], sensitivity was 63% [42.4-80.6], specificity was 88.9% [80-94.8%], and odds ratio was 13.6 [4.8-38.6].

**Disclosures:**

**Eric Pamer, MD**, Seres Therapeutics: Inventor on patent application # WPO2015179437A1 and #WO2017091753A1 and receives royalties from Seres Therapeutics, Inc.

